# Development and evaluation of the Flourish Diabetes Programme

**DOI:** 10.4102/hsag.v30i0.2923

**Published:** 2025-04-04

**Authors:** Sylvia Kruger, Elmari Deacon, Esme van Rensburg, David Segal

**Affiliations:** 1 Department of Psychology, Faculty of Health Sciences, Community Psychological Research, North-West University, Potchefstroom, South Africa; 2 Department of Psychology, Faculty of Health Sciences, Optentia Research Unit, North-West University, Vanderbijlpark, South Africa

**Keywords:** adjustment to closed-loop technology, closed-loop technology, Delphi method, intervention, positive psychology, type 1 diabetes

## Abstract

**Background:**

Adolescents with type 1 diabetes face unique challenges in adapting to closed-loop technology. Positive psychology interventions may aid in promoting psychological adjustment, enhancing well-being and supporting the effective use of this technology.

**Aim:**

This study describes the development, content and evaluation of a positive psychology intervention, the Flourish Diabetes Programme, to facilitate adjustment to closed-loop technology among adolescents with type 1 diabetes.

**Setting:**

The Flourish Diabetes Programme is an online, interactive intervention aimed at adolescents living with type 1 diabetes who use closed-loop technology.

**Methods:**

The development of the programme was informed by qualitative research with adolescents, qualitative document analysis and the design and development model. The Delphi method was used to evaluate the intervention, where 11 experts provided feedback to refine and enhance the programme.

**Results:**

Feedback from the Delphi panel informed the refinement and finalisation of the Flourish Diabetes Programme, contributing to an evidence-based and user-centred intervention.

**Conclusion:**

The Flourish Diabetes Programme is a tailored online resource designed to support adolescents in adapting to closed-loop technology. Insights from the Delphi panel helped shape the programme into a practical tool for promoting positive adjustment.

**Contribution:**

This intervention is the first step in developing effective, evidence-based resources to help adolescents with type 1 diabetes manage new medical technologies, specifically closed-loop systems, using positive psychological principles.

## Introduction

Type 1 diabetes is an autoimmune disease, which can occur at any age, although it is mostly diagnosed in youth (Harjutsalo, Sjöberg & Tuomilehto 2008). Type 1 diabetes cannot be prevented and can lead to complications, if untreated (Messer 2019). The treatment of type 1 diabetes requires complex self-management behaviours and exogenous insulin for life (O’Connor et al. 2018). Technology plays an integral role in the lives of adolescents living with type 1 diabetes (Pals et al. 2021), yet there exists a notable inconsistency in the adoption and utilisation of diabetes technology within this age group when compared to younger children and adults. Despite advancements, adolescents need targeted support to adopt diabetes technology, tailored specifically to the unique challenges faced by adolescents in utilising diabetes technology (Jaser et al. 2020).

**FIGURE 1:**
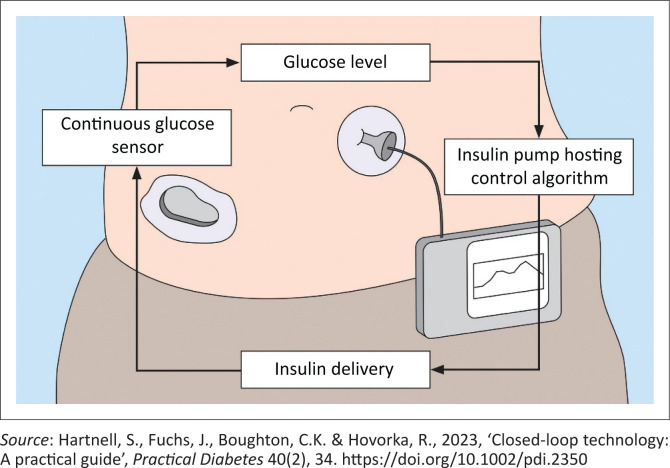
Diagram of how a closed-loop insulin delivery system works.

Closed-loop technology, often referred to as an ‘artificial pancreas’, integrates a continuous glucose monitor (CGM) with an insulin pump that automatically adjusts basal insulin based on real-time glucose readings. While the system automates some aspects of diabetes management, meal boluses still require manual administration (Forlenza, Buckingham & Maahs 2016). Figure 1 is a depiction of a closed-loop system.

A CGM measures glucose levels and sends the data via Bluetooth to a control system on an insulin pump or smartphone. The system calculates how much insulin is needed and tells the pump to inject fast-acting insulin under the skin. It adjusts insulin delivery every 5 min (Figure 1).

As the closed-loop system only automates parts of diabetes care, psychological factors are an important consideration in the use of such a system, because ultimate success with the system depends on the engagement of the person using the technology (Adams et al. 2018). Utilising diabetes technology to its full potential also requires education on the closed-loop system (Rabbone et al. 2022). Despite improved glucose control, numerous psychological barriers exist that may limit the benefits of closed-loop technology (Forlenza et al. 2018). Examples of barriers to the adoption of diabetes technology are diabetes distress (Tanenbaum et al. 2017), wearing the device on one’s body, disappointment in glucose control (Messer et al. 2020), a feeling of being overwhelmed by the amount of data provided by the closed-loop system (Kubiak et al. 2020), attitudes and beliefs regarding the technology and unrealistic expectations of the system (Borges & Kubiak 2016). When psychological barriers are considered, it is crucial that psychosocial factors are addressed and improved to increase the acceptance of closed-loop systems (Barnard et al. 2015). Considering the social and emotional aspects of living with diabetes, technology is crucial for healthcare professionals and researchers (Pals et al. 2021) to facilitate optimal outcomes (Kubiak et al. 2020).

Limited studies have investigated the psychological impact of diabetes technology, for example the impact thereof on people’s well-being or quality of life (Weissberg-Benchell, Antisdel-Lomaglio & Seshadri 2003), and little is known about the psychological factors that improve the usage of diabetes technology (Borges & Kubiak 2016). Medically, closed-loop systems can improve one’s HbA1c (haemoglobin A1c) and reduce hyperglycaemia (Beato-Víbora et al. 2021; Tauschmann et al. 2019). On a psychological level, improvement in diabetes-specific emotional distress is associated with the use of diabetes technology (Vesco et al. 2018), and it has been found that emotional well-being improves with the amount of time spent in the target blood glucose range (time in range) (Rabbone et al. 2022).

Diabetes management during adolescence is especially challenging (Hilliard et al. 2017), and despite the increased use of diabetes technology, most adolescents do not meet the recommended treatment targets (Jaser et al. 2020). Managing diabetes as an adolescent involves a complex interaction of medical, behavioural and social changes, where psychological functioning is a crucial component (Gonzalez, Tanenbaum & Commissariat 2016). On a psychological level, negative emotions linked to diabetes self-management are related to poor glucose control (Shapiro et al., 2018). On the contrary, positive psychology focusses on well-being, strength and optimal functioning (Seligman & Csikszentmihalyi 2000).

With regard to adolescents living with type 1 diabetes, no positive psychology intervention has specifically focussed on their adjustment to closed-loop technology although various research studies have explored interventions in the context of health and positive psychology (i.e. Charlson et al. 2007; Jaser et al. 2020; Ogedegbe et al. 2012; Peterson et al. 2012). Hilliard, Powell and Anderson (2016) argue that an emphasis on positive psychology interventions in type 1 diabetes (e.g. Jaser et al. 2014) can be beneficial, as such interventions would focus on the strengths of the adolescents and their families to promote resilience. It is suggested that positive affect can have a positive influence in conditions, such as diabetes, where behavioural factors play a role (Pressman & Cohen 2005). Positive emotion, engagement, relationships, meaning and accomplishment (PERMA), as a positive psychology model, was used in the development of the intervention in this study. The PERMA model of well-being focusses on positive emotions, engagement, relationships, meaning and accomplishment (Seligman 2011). Seligman (2011) argues that in optimal human functioning and fulfilment, and as a result of the components of the PERMA model, flourishing is achieved.

The second important theory used in the development of the intervention was the unified theory of acceptance and use of technology (UTAUT). This theory is relevant in the context of adjustment to closed-loop technology and emphasises the important role of the individual’s beliefs, attitudes and perceptions in making decisions in healthcare and using technology (Venkatesh & Davis 2000). According to the UTAUT model, effort expectancy, performance expectancy, social influence and facilitating conditions are the most important factors of behavioural intention (Venkatesh et al. 2003).

This article details the Flourish Diabetes Programme’s development and evaluation.

## Research methods and design

### Design

Within the framework of intervention research, the steps of Thomas and Rothman’s (1994) design and development model were utilised when the intervention programme was compiled. This model has six phases: problem analysis and project planning; information gathering and synthesis; intervention design; early development; evaluation and advanced development and the dissemination of the intervention (De Vos & Strydom 2011; Thomas & Rothman 1994).

**FIGURE 2:**
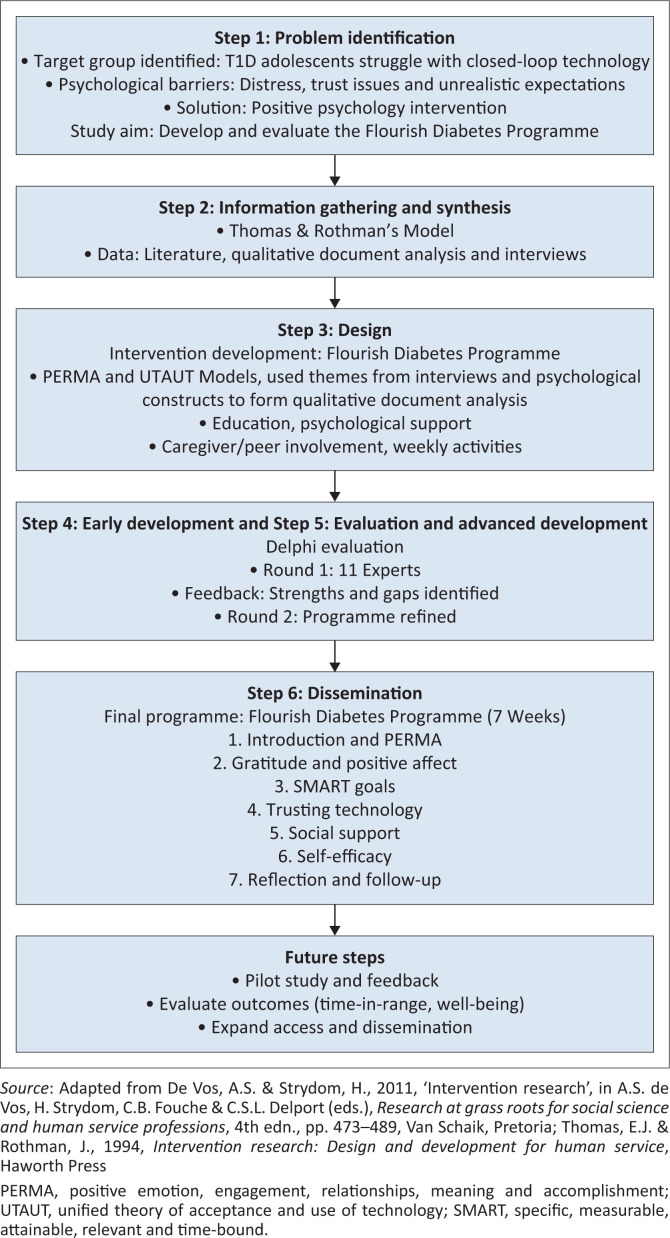
Development and evaluation of the Flourish Diabetes Programme (The steps of intervention research that guided the study).

The steps of Thomas and Rothman’s (1994) design and development model as well as the integration of a previous phenomenological study and qualitative document analysis by the current authors, the PERMA model and the UTAUT are summarised in Figure 2 and are discussed further within this section.

The authors have previously conducted a phenomenological study with adolescents using closed-loop technology and a qualitative document analysis (Kruger et al. 2023, 2024). These studies provided the basis for the proposed intervention programme. The aim of the phenomenological study was to obtain, by using interviews, an in-depth understanding of the lived experiences of adolescents who had successfully adjusted to closed-loop technology. The aim of the qualitative document analysis was to identify aspects that are important in people’s effective adjustment to living with closed-loop technology.

In the phenomenological study and the qualitative document analysis, themes were identified to identify essential constructs of the intervention programme. The identified themes were the importance of knowledge and diabetes education, the process of positive adjustment to closed-loop technology, a positive outlook and building a relationship with diabetes. These themes were used to inform the content and structure of a preliminary intervention programme. The findings are reported in a preceding article by Kruger et al. (2023). In addition, the researchers built on the work of Cohn et al. (2014); DuBois et al. (2016); Gonzalez et al. (2016); Jaser et al. (2014); Jaser et al. (2020); Loseby et al. (2021) and Schache, Hofman and Serlachius (2019), as well as Seligman’s (2011) PERMA framework as the building blocks of well-being and the UTAUT (Gonder-Frederick et al. 2016; Venkatesh & Davis 2000).

A preliminary positive psychology intervention programme was developed with the objective to assist adolescents diagnosed with type 1 diabetes to successfully adjust to closed-loop technology. After that, a qualitative Delphi study was conducted to determine the structure and content of the Flourish Diabetes Programme. The Delphi method is a consensus technique (Trevelyan & Robinson 2015) that is useful in the context of health care (Keeney, Hasson & Mckenna 2011), especially in the development of guidelines and the identification of key constructs of interventions (Lai et al. 2015; Ward et al. 2014). The Delphi method is a structured communication process. It was used to achieve expert consensus on the key components of the intervention in order to improve engagement with the technology. Experts in the field of diabetes were invited to participate in the study with the aim of establishing consensus on the final intervention programme. The perspectives of the professionals assisted the researchers in refining the intervention programme.

### Setting and participants for the Delphi study

Experts, defined as specialists in their field (Keeney et al. 2011), were purposively and snowball-sampled, resulting in the selection of 11 diabetes professionals. They received email invitations detailing the study, participation expectations, confidentiality measures and withdrawal rights. Anonymity was ensured to encourage open and diverse opinions. This study adhered to the *Protection of Personal Information Act* (POPIA) to ensure confidentiality and secure handling of participant data.

This study included a diverse group of experts – endocrinologists, general practitioners, diabetes educators, dietitians and psychologists – ensuring heterogeneity (Keeney et al. 2011). Participants needed at least 2 years’ experience, work with adolescents and be professionally registered. Eleven experts joined Round 1, and 10 continued in Round 2 because of one expert’s withdrawal. Table 1 summarises their details.

**TABLE 1:** Participants’ particulars for the Delphi panel.

Pseudonyms	Professional registration	Years of experience	Context of work
Odette	Dietitian	18	Academic and private practice
Hannah	Dietitian	28	Public hospital
Kayla	Dietitian	10	Public hospital
Natalie	Dietitian and diabetes educator	13	Private practice
Emma	Dietitian and diabetes educator	11	Private practice
Sophy	Registered nurse and diabetes educator	25	Private practice
Mia	Registered nurse and diabetes educator	28	Academic and private practice
Samuel	Psychologist	6	Private practice
Jack	Paediatric endocrinologist	10	Private practice
Wayne	Paediatric endocrinologist	35	Academic and private practice
Lucy	General practitioner	23	Private practice

### Data collection for the Delphi study

A modified online Delphi technique was utilised. The data collection consisted of two rounds and included 11 experts. A modified online Delphi technique was used, consisting of two rounds with 11 experts in adolescent type 1 diabetes care, whose experience ranged from 6 to 35 years. The number of rounds depended on achieving consensus (Hsu & Sandford 2007). In Round 1, experts received a programme outline, website link and open-ended questionnaire, with 2 weeks to respond. The Flourish Diabetes Programme materials included educational content on diabetes and closed-loop technology, weekly activities over 7 weeks and a forum for adolescent feedback, with caregiver involvement and three facilitation sessions. The programme focussed on PERMA principles and covered self-efficacy, trust in technology, motivation, goal-setting, positive affect and support.

The questionnaire sent to the experts made use of open-ended questions to allow for detail and richness in the participants’ responses. The open-ended questionnaire asked for information regarding the expert’s experience in the field of diabetes and their evaluation of the proposed intervention programme in terms of its content and structure.

In the first round, participants answered questions on adolescents’ challenges with closed-loop technology, difficulties in diabetes management, psychological factors aiding adjustment and essential skills for success. They evaluated the intervention’s relevance, suggested additions or removals, assessed the structure and content, engagement strategies and strengths and weaknesses of the Flourish Diabetes Programme, providing recommendations for improvement.

The programme was revised based on Round 1 feedback. In Round 2, experts received a summary of anonymised responses to encourage honest input (Keeney et al. 2011). They were asked to evaluate the updated programme and suggest any further improvements.

### Data analysis for the Delphi study

The data obtained from the Delphi technique were analysed by means of thematic analysis (Braun & Clarke 2020) with the aim of obtaining a thorough understanding of the essential constructs that should be included in the intervention programme and the structure of the programme. Following the thematic analysis, adjustments were made to the proposed intervention. The following process was followed for both rounds: Firstly, the researchers familiarised themselves with the data by reading and re-reading the feedback from the experts. Secondly, they generated codes for important features of the data relevant to the research question that guided the analysis. Thirdly, they searched for possible themes by identifying similarities in the codes. Fourthly, they reviewed the generated themes with regard to the codes and the transcripts. Fifthly, they named the final themes and developed a description of each theme. The final step was producing the final report and disseminating the findings. The thematic analysis continued until the research question had been answered and no new themes emerged (Willig 2013).

### Measures of trustworthiness

The researchers ensured theme validity by continuously referencing raw data, using verbatim extracts and maintaining a reflective journal to minimise bias. A co-coder verified interpretations, and verbatim quotations supported findings. The lead researcher, a trained psychologist, ensured accurate analysis.

### Ethical considerations

The study was approved by the Health Research Ethics Committee (HREC) of the North-West University, South Africa (reference no.: NWU-00266-21-A1). Informed consent was obtained both verbally and in writing from all participants.

## Results from the Delphi panel

Seven themes emerged and were incorporated into refining the intervention programme. The seven themes are discussed in this section.

### The content is helpful in the process of adjustment

Experts found the intervention valuable for adolescent adjustment to closed-loop technology and diabetes identity. Strengths highlighted included positive affect and gratitude (Jack, paediatric endocrinologist), goal-setting (Lucy, general practitioner), group support (Samuel, psychologist), coping skills (Hannah, dietitian), psychological components and problem solving (Wayne, paediatric endocrinologist), weekly tasks (Mia, nurse and diabetes educator) and practical skills (Emma, dietitian and diabetes educator).

Mia and Emma noted a gap in emotional support in diabetes training, with trust, motivation and positive goal setting identified as key for adjustment. Emma called the psychological aspect one of the most overlooked parts of diabetes care. Hannah described the programme as structured, organised and easy to follow, while Lucy praised its well-thought-out content. Emma and Hannah agreed the intervention would benefit a wider diabetes population.

### Caregiver and peer involvement

All the experts were of the opinion that the amount of involvement from caregivers within the intervention was adequate and would give the adolescents a sense of autonomy. In this regard, Emma (dietitian and diabetes educator) stated: ‘Transfer of care from parent or guardian to the patient is a difficult journey and including them in some of the intervention components will be valuable’. The experts emphasised the importance of engaging with other adolescents to have a sense of connection and support and possibly providing practical support to one another. Samuel (psychologist) stated that ‘the opportunity to engage in the group process and to have a sense of connection will be incredibly therapeutic’. All the experts viewed the support from caregivers, the support from the peer group and the weekly feedback as beneficial. It was highlighted that, considering adolescents’ developmental needs, during this phase, there is an increased need for independence, with some parental support. In this regard, Sophy (registered nurse and diabetes educator) remarked that ‘the intervention should align with the academic, intellectual and most importantly, the emotional abilities of the participants’.

### Weekly feedback

All the experts viewed the weekly feedback as beneficial and a means of maintaining engagement with the intervention. Natalie (dietitian and diabetes educator) especially highlighted the importance of the weekly feedback. It was emphasised that the weekly feedback was appropriate in terms of the amount of time required by the adolescents to provide feedback. Emma (dietitian and diabetes educator) stated that ‘the forum is a great way to solidify what was learnt’, while Sophy (registered nurse and diabetes educator) described the weekly feedback as ‘giving you an idea in which direction the intervention is going’.

### Identify barriers at onset

Samuel (psychologist) identified diabetes distress as a potential barrier to engagement with the intervention and suggested including a screening tool prior to participation in order to address any potential barriers. Sophy (registered nurse and diabetes educator) also suggested that adolescents should attend the programme with adolescents who are using the same diabetes technology in order for the adolescents to relate to one another and avoid confusion. During the second round, the other experts commented that this was a useful suggestion.

### Technical feedback

Recommended adjustments included technical aspects and using language in a sensitive manner. For example, Odette (dietitian) and Emma (dietitian and diabetes educator) suggested changing the word ‘parent’ to ‘caregiver’, and Odette suggested changing the word ‘task’ to ‘activity’. Sophy (registered nurse and diabetes educator) also suggested aligning the content to the South African context, for example referring to mmol/L instead of mg/dL. Natalie (dietitian and diabetes educator), Samuel (psychologist) and Wayne (paediatric endocrinologist) recommended that a form of incentive should be used to maintain engagement by the adolescents; accordingly, ‘badges’ were created on the website that adolescents could earn after participating in the activities of each week.

### Recommendations regarding the content

Experts identified gaps in the educational content. Hannah (dietitian) recommended adding healthy living information, while Wayne (paediatric endocrinologist) suggested removing restrictive diabetes guidelines. Odette (dietitian) called for more substance in nutrition and self-management and Samuel (psychologist) proposed using videos. In response, general diabetes guidelines and video content were added. Emma (dietitian and diabetes educator) praised the content flow and emphasised giving adolescents a platform to share challenges and solutions. She and Kayla (dietitian) also recommended replacing collage making with social media-based activities for better adolescent engagement.

### Duration of the programme

Most experts found the programme duration adequate, though Jack (paediatric endocrinologist) worried it might be too time intensive. To address this, weekly time requirements were clarified to ensure integration without overload. Hannah (dietitian) noted the tasks were concise and manageable. Samuel (psychologist), Mia (nurse and diabetes educator) and Natalie (dietitian and diabetes educator) suggested gathering adolescent feedback post-programme and holding a 3-month follow-up to assess lasting changes and identify barriers.

## Discussion

The Flourish Diabetes Programme was informed through a qualitative document analysis, a phenomenological study and a Delphi study. The data obtained from these studies were used to inform the final intervention programme in terms of its content and structure. The seven themes from the Delphi study and the recommended adjustments and strengths identified by the experts were built on and incorporated into the final programme. The experts were of the opinion that the proposed intervention programme would assist adolescents in their adjustment to closed-loop technology. The importance of support was highlighted by the experts throughout the programme, and this aspect was built on in the development of the final intervention programme. This is in line with the substantial amount of evidence corroborating the beneficial impacts of peer support (Pienaar & Reid 2021; Qi et al. 2015).

With regard to the method of delivery, the experts pointed out that the weekly feedback should be emphasised in order to maintain participant engagement. It was also suggested that potential barriers should be identified before starting with the intervention programme. Regarding the content, the experts recommended the inclusion of more general information on diabetes, as well as video content, to make the programme more suitable for the developmental level of adolescents and to ensure that the amount of time needed to engage in the programme on a weekly basis was not too much.

The experts commented that the researchers should be mindful of using language in a sensitive manner, in line with Dickinson et al. (2017). For example, using the word ‘caregiver’ instead of ‘parent’ was suggested. Other recommendations were that the developmental level of adolescents should be kept in mind, and an incentive should be used to encourage active participation.

Obtaining feedback from experts in various fields of diabetes was beneficial, as the researchers could draw from their diverse opinions and backgrounds. Thus, it assisted with the refinement of the intervention programme in a holistic manner.

Technological advances are leveraged in this programme as the intervention content will be delivered through a free website, making it more accessible to participants. The researchers hope this programme will be a valuable addition to the medical treatment of adolescents living with type 1 diabetes by aiding their adjustment to closed-loop technology.

### Final intervention programme after expert review

It is noteworthy that most of this section is written in the present and future tense because the programme has been developed and evaluated, but it has not yet been implemented.

### General

The Flourish Diabetes Programme is an online, interactive website designed for adolescents between the ages of 15 and 18 years who are living with type 1 diabetes and using closed-loop technology. The name of the programme – Flourish Diabetes Programme – is derived from the work of Seligman (2011). This age group was chosen because adolescents of different ages navigate different challenges, developmental tasks and changes on their journey with diabetes. Consequently, within the intervention, it will be beneficial for adolescents of the same age group to learn from one another. The duration of the programme is 7 weeks, in line with previous positive psychology interventions, which usually last 4 to 8 weeks (Bolier et al. 2013).

### Support

As support was identified as a crucial construct of living positively with diabetes (Ingersgaard et al. 2019; Montali et al. 2022), caregivers will be involved during parts of the programme. During each week of the programme, some interaction between caregivers and adolescents will be facilitated. Peer support will also be facilitated during the course of the programme.

### Structure

Upon signing up, participants receive the Problem Areas in Diabetes – Teen (PAID-T) screening tool via email to assess diabetes distress, a potential barrier to engagement. Those with high distress may opt for a short online meeting with a facilitator. Participants also indicate their closed-loop system to ensure they are grouped with others using the same technology, reducing confusion. The PAID-T is repeated post-programme to assess improvements.

Programme content and weekly activities will be available on a website, reinforcing PERMA principles over 7 weeks. The site includes educational materials on PERMA, time in range, glucose control, nutrition and diabetes burnout. Each week, caregivers and adolescents read content, complete positive psychology exercises and participate in weekly caregiver activities. The programme features three online facilitation sessions, email or SMS reminders and a forum for experience-sharing, encouraging engagement. Participants earn badges as incentives for completing activities.

### Content and constructs

Table 2 provides a summary of the programme and weekly content.

**TABLE 2:** Summary of Flourish Diabetes Programme.

Week	Aim	Content
1	Introduction to the Structure of the Intervention Programme and the Concept of Well-Being and PERMA (Facilitation Session)	Icebreaker activity and introduction to well-being and PERMA concepts. Online group facilitation session providing orientation Caregivers are part of the online session Engage in individual and group activities to elicit positive emotions. Caregivers were asked to do one activity with adolescents to elicit positive emotions
2	Positive Affect and Gratitude	Daily reflection on gratitude elements and sharing type 1 diabetes facts Reflect on gratitude and share diabetes knowledge.
3	Motivation and Positive Goal Setting	Setting SMART goals and completing the circle of control activity Set achievable goals and focus on controllable factors
4	Learning to Trust the Technology	Reviewing glucose data and reframing perspectives. Interpret glucose data and develop trust in technology
5	Support (Facilitation Session)	Sharing positive social media posts, discussing role models and discussing support systems. Spend quality time with someone the adolescents value. Engage in act of kindness.
6	Self-Efficacy and Responsibility	Completing the ‘best possible self’ activity and identity steps to achieve goals Caregiver-adolescent meeting Foster responsibility and independence in diabetes management
7	Tips on Continuing With the above skills beyond the Intervention (Facilitation Session)	Reflecting on programme experience and creating social media posts on what was learned during the programme Reflect on learned skills and share experiences PERMA wheel Caregivers share what they are proud of

SMART, specific, measurable, attainable, relevant, time-bound; PERMA, positive emotion, engagement, relationships, meaning and accomplishment.

### Week 1: Introduction to the structure of the intervention programme and the concept of well-being and PERMA (facilitation session)

Week 1 introduces participants to the programme structure through an online facilitation session led by a diabetes educator or psychologist. The session includes introductions, questions and an overview of the 7-week programme, with weekly reminders and positive psychology exercises via email. Caregivers join initially to understand their role, then step back to promote adolescent independence.

The session features an icebreaker, a discussion on well-being and PERMA and assistance with website sign-up. Participants share positive experiences and aspects of closed-loop technology. Weekly tasks include individual and social activities to elicit positive emotions, with caregivers encouraged to participate. Caregivers complete one shared activity with the adolescent during Week 1.

### Week 2: Positive affect and gratitude

In Week 2, participants are introduced to the power of gratitude in cultivating a positive mindset. Central to this week is the acronym ‘GLAD’ which stands for Gratitude, Learning, Accomplishment, and Delight. This simple yet effective framework guides participants in reflecting on daily positive experiences, helping shift focus from challenges to strengths. Each day, adolescents are encouraged to journal about one thing they are grateful for, something they learned, an accomplishment they achieved (no matter how small), and a moment of delight or joy. These reflections foster emotional resilience and build a habit of noticing the good amidst the difficulties of managing type 1 diabetes. To support this process, the programme website provides prompts and examples to make the journaling accessible and engaging. In addition, adolescents and their caregivers are invited to share one new fact about type 1 diabetes with each other daily, promoting shared learning, empathy, and stronger communication within families.

### Week 3: Motivation and positive goal setting

Week 3 focusses on positive goal setting using SMART goals (specific, measurable, attainable, relevant and time-bound) to promote autonomy and accountability (Nguyen-Vaselaar & Lappe 2021). Adolescents set a weekly diabetes-related SMART goal, track progress daily and share it with their caregiver, who provides support and feedback. Participants also complete the Circle of Control activity (Covey 2020) to shift focus to controllable factors, avoiding fixation on external challenges. Examples and resources are available on the programme website to guide engagement.

### Week 4: Learning to trust the technology

In Week 4, participants engage in activities aimed at understanding and utilising glucose data from closed-loop technology. They are prompted to download their glucose data to visualise trends, while caregivers and adolescents read an article on interpreting these data, empowering them to utilise it effectively. The goal is to enhance understanding and trust in the closed-loop system. Adolescents apply their newfound knowledge to their own data, while caregivers solicit feedback on its application. Additionally, participants explore reframing strategies for interpreting diabetes data positively, focussing on informed decision-making rather than success or failure. This reading fosters a constructive mindset towards diabetes management.

### Week 5: Support (facilitation session)

Week 5 features an online facilitation session aimed at promoting support and positivity. Participants prepare by finding and sharing uplifting content on social media related to living positively with type 1 diabetes. They then engage in discussions about meaningful relationships and offer advice to those newly diagnosed with diabetes. Psychoeducational content focusses on utilising support systems effectively, addressing practical challenges with closed-loop technology, and encouraging open dialogue within the group. Caregivers are prompted to offer positive affirmations to adolescents, while adolescents are encouraged to spend quality time with loved ones and perform acts of kindness.

### Week 6: Self-efficacy and responsibility

During Week 6, the focus shifts to fostering independence and responsibility in diabetes management, aligning with the critical developmental stage of adolescence (Chiang et al. 2014). Caregivers receive guidance via email on supporting this transition and adapting to the changing dynamics of their relationship with adolescents. Participants engage in a ‘best possible self’ activity (Sheldon & Lyubomirsky 2006) to envision a positive future, setting health behaviour goals and identifying steps towards achieving them. Caregivers and adolescents are prompted to hold a caregiver-adolescent meeting, where they share positive affirmations and discuss ways to improve their diabetes management and relationship dynamics. Requests made during the meeting aim to enhance mutual understanding and support, fostering a collaborative approach to diabetes care.

### Week 7: Tips on continuing with the above skills beyond the intervention (facilitation session)

Participants are directed to download Mead’s (2023) PERMA wheel worksheet from the programme website ahead of the final facilitation session. They receive a brief overview of the PERMA model’s constructs and are prompted to rate their satisfaction with each aspect of their life on the wheel. During the session, participants reflect on their programme experience, discuss how they will apply newfound skills and identify areas for improvement within the PERMA model. Continuous use of the PERMA wheel is encouraged for self-assessment and ongoing integration of model aspects. Caregivers join part of the session to express pride in adolescents’ progress. The final activity entails creating a social media post about programme learnings or their diabetes journey, fostering motivation and reflection. Feedback from participants is gathered to refine the programme, with a follow-up meeting held 3 months after completion of the programme to assess sustained changes and address any barriers encountered. The feedback will be in the form of individual interviews with a focus on the satisfaction of closed-loop technology and its integration into daily life as well as support received from caregivers. It is also recommended that a pilot study of the current programme takes place where quantitative measures are taken into account, for example, time in range and HbA1c.

## Implications

While diabetes is a part of the focus of the programme, the programme aims to view adolescents holistically and to provide them with skills they can use in their daily lives, not only within the context of diabetes. Healthcare professionals can also use the constructs and exercises employed within this programme to help adolescents adjust to living with closed-loop technology. Psychological barriers, including diabetes-related stigma, anxiety about technology dependence and concerns over device visibility, significantly impact engagement with closed-loop systems. It is possible that stigma can deter adolescents from consistent technology use, while anxiety over hyperglycaemia and hypoglycaemia can lead to excessive glucose monitoring and fear-based decision-making. Addressing these barriers within interventions is crucial in order to improve adherence and long-term health outcomes.

## Limitations

Although the proposed intervention programme is grounded in empirical data – a literature review – and the principles of positive psychology and has been evaluated by a panel of experts, the programme has not been pilot tested. A recommendation for further study is to pilot test the programme to assess its uptake, feasibility and effectiveness. Because the programme has not yet been pilot tested, it is important to gather feedback from adolescents living with diabetes who use closed-loop technology. This feedback will help refine the programme, taking into account the developmental levels and interests of adolescents. In addition, it is recommended that further feedback from mental health experts is obtained as only one mental health expert agreed to participate in the current Delphi study. Lastly, motivation from adolescents should be considered, and it is possible that some participants would not want to complete the full 7 weeks. During the programme’s design, the authors aimed to strike a balance between providing engaging content and ensuring the programme’s manageability within adolescents’ schedules. However, the potential for sustaining adolescents’ motivation will be investigated in a pilot study. If this proves to be a challenge, adjustments to the programme will be considered. Future iterations of this research may consider incorporating perspectives from community leaders, such as religious representatives, who may provide additional emotional support and facilitate participant comfort in research settings.

## Conclusion

This article described the development, content and evaluation of the Flourish Diabetes Programme, which was designed to assist adolescents with their adjustment to closed-loop technology. The intervention development process, the content of the intervention and the evaluation of the programme were described. The Delphi method was used to evaluate the proposed intervention programme by utilising a panel of experts to refine the programme in terms of its content and structure. The feedback received from the experts indicates that the Flourish Diabetes Programme has the potential to benefit adolescents in their adjustment to closed-loop technology. The different fields of diabetes in which the experts work assisted the researchers in gaining a broad understanding of adolescents’ adjustment to closed-loop technology and refining the programme to provide a holistic programme for adolescents.
